# Characterization of Virulence Factors, Cellular Stress Response, and Antifungal Susceptibility Testing of *Trichosporon* spp. Isolated from Northeast Brazilian Patients

**DOI:** 10.3390/jof11040255

**Published:** 2025-03-26

**Authors:** Márcia Gabriele de Souza Jimenez, Matheus Firmino de Azevedo, Elaine Cristina Francisco, Ana Maria de Andrade Oliveira Boczar, Ana Carolina Barbosa Padovan, Eveline Pipolo Milan, Walicyranison Plinio da Silva Rocha, Guilherme Maranhão Chaves

**Affiliations:** 1 Laboratório de Micologia Médica e Molecular, Departamento de Análises Clínicas e Toxicológicas, Universidade Federal do Rio Grande do Norte, Natal 40301-110, RN, Brazil; marciagaby15@gmail.com (M.G.d.S.J.); matheusfirmino2008@hotmail.com (M.F.d.A.); 2 Laboratório Especial de Micologia, Disciplina de Infectologia, Universidade Federal de São Paulo, São Paulo 04039-032, SP, Brazil; elaineperol@yahoo.com.br; 3 Departamento de Micologia, Universidade Federal de Pernambuco, Recife 52051-380, PE, Brazil; andrade.oliveira@ufpe.br; 4 Departamento de Microbiologia e Parasitologia, Universidade Federal de Alfenas, Alfenas 37700-000, MG, Brazil; carolpadovan.pesquisaclinica@gmail.com; 5 Departamento de Infectologia, Universidade Federal do Rio Grande do Norte, Natal 40301-110, RN, Brazil; evepipolo@gmail.com; 6 Departamento de Ciências Farmacêuticas, Universidade Federal da Paraíba, João Pessoa 58051-900, PB, Brazil; wps@academico.ufpb.br

**Keywords:** *Trichosporon* spp., genotyping, virulence, cellular stress, antifungal susceptibility, trichosporonosis

## Abstract

*Trichosporon* spp. are emerging pathogens that may cause high mortality rates, specifically among immunocompromised individuals. The objectives of this study were to perform a phylogenetic analysis of *Trichosporon* spp. clinical isolates. We also evaluated the expression of different virulence factors in vitro. In addition, the isolates were grown in the presence of cell-wall and membrane stressors. The antifungal susceptibility profiling was determined. The most prevalent strains belonged to the recently described species *T. austroamericanum*, with 17 isolates. The other strains were identified as follows: *T. asahii* (n = 4), *T. faecale* (n = 2), and *T. asteroides* (n = 2). All the isolates of *T. asahii*, *T. faecale*, and *T. asteroides* were strong biofilm producers. Statistical analysis revealed that *T. asahii* strains produced more biofilm than *T. austroamericanum*. Higher cell surface hydrophobicity was also found for *T. asahii* isolates compared to *T. austroamericanum* counterparts. *T. austroamericanum* seems to be more susceptible to Congo Red, Calcofluor White, and SDS than *T. asahii*. It is possible to conclude that *Trichosporon* spp. may present peculiarities in terms of the expression of different virulence factors in vitro, besides displaying a variable susceptibility to different cellular stressors. *T. faecale* isolates may present high MICs to the azoles, while *T. asahii* against amphotericin B.

## 1. Introduction

*Trichosporon* spp. are yeast-like fungi belonging to the Division Basidiomycota, predominantly found in tropical and temperate zones and widely distributed in nature [1,2]. They can be isolated from water, soil, decaying wood, and bird and bat excrement. In humans, these microorganisms may belong to the gastrointestinal and oral cavity microbiota or transiently colonize the skin, nails, and vagina of healthy individuals [2,3,4,5,6].

*Trichosporon* spp. may show white-, beige-, or cream-colored colonies, with a dry, cerebriform, or radiated surface aspect when grown on Sabouraud Dextrose Agar (SDA) [2,7]. Microscopically, they show all the possible structures expected for a yeast, such as blastoconidia, pseudohypha, true hyphae, and arthroconidia [1,2,7,8]. Laboratory diagnosis of superficial and invasive trichosporonosis is based on direct examination of clinical samples on microscope slides containing KOH (10–40%) or even wet-mount and/or tissue biopsy specimens and culture findings. In addition, *Trichosporon* spp. are non-fermentative of carbohydrates and able to degrade urea [4].

*Trichosporon* spp. may cause superficial mycoses, these being strongly associated with white *piedra*, a fungal infection characterized by the presence of hyaline nodules of mucilaginous consistency that adhere to the extrafollicular region of the hair shaft, being also found on pubic hair, beard hair, mustache hair, armpit hair, and eyebrows [4,9,10].

In addition to harmless superficial infections, *Trichosporon* spp. may cause severe systemic diseases, especially in patients with hematological malignancies, tumors, neutropenia, and the presence of a central venous catheter. Disseminated trichosporonosis strains are potentially severe and associated with a high mortality rate (of up to 50–80%) when related to hematological diseases or neutropenia [4,6]. These microorganisms may also cause pneumonia, endophthalmitis, meningitis, urinary tract infection, and hypersensitivity pneumonitis [11,12,13,14]. According to data from the ARTEMIS DISK collection, *Trichosporon* spp. are considered the second or third non-*Candida* yeast isolated in clinical laboratories [2,13,14,15].

The sequencing of the *IGS1* region of the rDNA has the best discriminatory power for *Trichosporon* spp. identification since the internal transcribed spacer region (ITS) is only 1% divergent among closely related species [16,17]. In 2015, a taxonomic reclassification of the genus *Trichosporon* resulted in the reassignment of some medically relevant species to other fungal genera, such as *Apiotrichum* and *Cutaneotrichosporon* [18].

The most clinically relevant *Trichosporon* species include *T. asahii*, *T. inkin*, *T. faecale*, and *T. ateroides* [3,19]. Recently, *T. austroamericanum* has been described as a novel species, closely related to *T. inkin*. The authors evaluated morphological, physiological, and molecular characteristics—including IGS1 rDNA sequencing and Amplified Fragment Length Polymorphism (AFLP) fingerprinting—and considered it a separate *Trichosporon* species [20].

*Trichosporon* spp. are able to express some virulence factors, including the following: the ability to grow at 37 °C (thermotolerance); adhesion to human epithelial and endothelial cells; biofilm formation; the presence of glucuronoxylomannan (GXM, a cell-wall component that is strongly antigenic); and the secretion of extracellular enzymes (phospholipases, proteinases, and hemolysins) [4,21,22]. Nevertheless, the specific roles of virulence factors have been poorly investigated [23], specifically among non-*T. asahii* species.

There is an enormous variation in the antifungal susceptibility profiling of different *Trichosporon* spp. Therefore, accurate identification at the species level is crucial for the empirical and early use of appropriate antifungal drugs [6]. Superficial infections caused by *Trichosporon* spp., such as white *piedra*, may be easily treated with either topical or oral azole antifungals when associated with proper hygiene habits [24]. However, invasive infection therapy is still considered a challenge as there is no consensus about the recommended treatment [25,26]. Several studies report minimal therapeutic success with amphotericin B or fluconazole and this is related to the high minimal inhibitory concentrations (MICs) found in vitro for antifungal susceptibility testing using both drugs [27]. Nevertheless, newer triazoles remain very active against most *Trichosporon* isolates, especially voriconazole [28,29], while echinocandins have minimal or no activity against basidiomycetes [30,31].

The aims of the present study were to properly identify *Trichosporon* spp. clinical isolates obtained from patients with superficial and systemic trichosporonosis. In addition, we have investigated the expression of different virulence factors in vitro, susceptibility to cell-wall and membrane stressors, as well as to antifungal drugs used to treat trichosporonosis. We fully phenotypically characterized isolates belonging to the recently described novel species *T. austroamericanum*.

## 2. Materials and Methods

### 2.1. Strains Used in the Present Study

*Trichosporon* spp. superficial isolates (n = 20) were obtained from children attending day care with clinical signs of white *piedra*. In addition, five isolates were recovered from patients with systemic infections admitted at a tertiary hospital from February 2012 to December 2018. Both institutions are located in the city of Natal, Rio Grande do Norte, Brazil (single-center study) and belong to the culture collection of the Laboratory of Medical and Molecular Mycology (LMMM), Department of Clinical and Toxicological Analyses, Federal University of Rio Grande do Norte. Clinical samples were collected according to the protocols approved by the Research Ethics Committee of the “Liga Norte Riograndense Contra o Câncer”, approved under the number 3.769.085.

### 2.2. Strain Reactivation and Preliminary Identification

The isolates were stored at −80 °C in YPD containing 20% glycerol. Cells were defrosted on ice and 100 µL of each cell suspension was added to 5 mL of YPD liquid medium (dextrose 20 g/L, peptone 20 g/L, yeast extract 10 g/L) and incubated in a rotator shaker (TE-420, Tecnal^®^, Piracicaba, Brazil) at 35 °C, at 200 rpm, for 48 h, for the reactivation and verification of cell viability. Subsequently, 100 µL of the cell suspension was inoculated on the surface of SDA (Oxoid, Basingstoke, Hampshire, UK) containing chloramphenicol (0.05 mg/mL; Arifenicol^®^, Ariston, São Paulo, Brazil) using a Drigalsky loop, and further incubated at 37 °C ± 2 °C for 48 h. To check for purity, yeast colonies were subcultured on CHROMagar Candida^®^ medium (CHROMagarTM Candida, Difco, Franklin Lakes, NJ, USA) and the plates incubated at 37 °C ± 2 °C for 72 h. The preliminary identification (screening for the *Trichosporon* genus) was based on the characteristics of the yeast cells observed microscopically after cultivation on corn meal agar containing Tween 80, glucose absence of fermentation, and positive urease test [32]. *T. asahii* CBS2630 was used as a reference strain for the phenotypic identification and virulence factors expression in vitro at the genus level.

### 2.3. DNA Extraction

The isolates were grown on plates containing YPD agar (BD Difco, Franklin Lakes, NJ, USA) at 30 °C for 72 h. Genomic DNA was extracted using the PrepMan^®^ Ultra Sample Preparation Reagent (Applied Biosystems, Foster City, CA, USA), according to the manufacturer’s instructions. DNA concentration was determined by optical density at 260_nm_ and purity (protein contamination) from the optical density ratio (O.D.) 260/280_nm_ using a Qubit quantifier (QubitdsDNA HS Assay Kit, Life Technologies, Carlsbad, CA, USA).

### 2.4. PCR Assay and Sequencing of the IGS1 Region

Molecular identification was performed by sequencing the *IGS1* region of rDNA using the 26SF and 5SR primer pair as previously described [33]. Purified PCR products were sequenced using the ABI PRISM 3100 automated sequencer (Applied Biosystems, Foster City, CA, USA). The sequencing reactions included each of the primers mentioned and the BigDye Terminator reagent kit (Applied Biosystems, Foster City, CA, USA) employed according to the manufacturer’s instructions. For the sequencing of the *IGS1* region, a pair of primers was used, as follows: 26SF (5-ATCCTTTGCAGACGACTTGA-3) and 5SR (5-AGCTTGACTTCGCAGATCGG-3) [33]. Contigs were assembled based on two reads per isolate and edited using Phred-Phrap-Consed targeting a Phred score of >30 [34,35]. The consensus was compared with the sequences deposited in the NCBI database (https://ncbi.nlm.nih.gov, accessed on 23 December 2024) using the BLASTn tool (https://blast.ncbi.nlm.nih.gov/Blast.cgi, accessed on 23 December 2024) considering ≥98% of the percentage of identity and coverage with an E-value of <10^−5^. The sequences obtained per isolate were deposited in GenBank (https://www.ncbi.nlm.nih.gov/genbank/, accessed on 23 December 2024) under the following accession numbers: PQ189028-PQ189052.

### 2.5. Trichosporon spp. Molecular Identification and Genotyping

Accurate species identification was achieved through phylogenetic analyses carried out with MEGA X software v. 11 [36], employing the Neighbor-Joining method based on the Kimura two-parameter model, incorporating 1000 bootstrap pseudo-replicates and considering gap positions [37,38,39]. Intraspecific diversity among *Trichosporon* spp. was assessed with the reference genotype sequences of *T. asahii* and *T. faecale* deposited in GenBank [40].

### 2.6. Trichosporon spp. Inoculum Standardization

For the phenotypic characterization of the clinical isolates, the samples were initially grown in NGY medium. When cells were inoculated by wet-looping in this medium and incubated for 18–24 h under mechanical agitation at 200 rpm, at 30 °C, an inoculum of approximately 2 × 10^5^ cells/mL was produced [41].

### 2.7. Trichosporon spp. Adhesion to Human Buccal Epithelial Cells (HBECs)

To standardize the inoculum, *Trichosporon* cells were grown overnight in NGY broth and the absorbance was measured at O.D._600nm_. Samples of human buccal epithelial cells (HBECs) were collected from healthy and non-yeast-colonized volunteers with a sterile swab, rubbed for 2 min in the oral cavity, and transferred to 25 mL conic tubes containing 5 mL of PBS (Phosphate-Buffered Saline; NaCl 8 g/L; KCL 0.2 g/L; Na_2_HPO_4_ 1.44 g/L; KH_2_PO_4_ 0.24 g/L; pH 7.2), kept refrigerated until the moment of experimentation. Yeast and HBEC cell suspensions were centrifuged at 1200 g, 4 °C, for 5 min and washed three times with PBS with a pH of 7.4 buffer. The inoculum was standardized to 5 × 10^6^ cells/mL (yeast) and 5 × 10^5^ cells/mL HBECs. The two types of cells were mixed in equal proportions (100 µL of each suspension) in sterile glass flasks and incubated under mechanical agitation at 37 °C for 1 h. Subsequently, cells were fixed in 200 µL of 10% formalin (in PBS) and the number of yeast cells adhered by each HBEC was determined. One-hundred-and-fifty HBECs were counted using an optical microscope (Olympus CX21, Tokyo, Japan) at 400 times magnification. The assay was performed in triplicate [42].

### 2.8. Trichosporon spp. Biofilm Formation

For the biofilm formation, the methodology proposed by Jin et al. [43] with some modifications was used [13]. *Trichosporon* spp. strains were cultivated on SDA at 37 °C for 18 h. The isolates were inoculated into 5 mL of YNB medium (Yeast Nitrogen Base, Difco^TM^, Franklin Lakes, NJ, USA) containing 50 mM glucose (D-glucose monohydrate P.A., Kinetics, San Jose, CA, USA) and incubated overnight for 18 h at 200 rpm. After the incubation period, the samples were centrifuged at a speed of 5000 rpm for 5 min at room temperature (25 ± 2 °C; RT), and the pellet was washed twice in 5 mL of PBS under the same centrifugation conditions. The pellet was resuspended in 5 mL of PBS and the cell concentration adjusted to 10^7^ cells/mL, which is equivalent to an optical density, O.D._520nm_, of 0.38. For the adhesion phase, the cells were transferred to sterile 96-well polystyrene microtiter plates (Cralplast, Sao Paulo, Brazil). One-hundred microliters of the suspension was added to each well and the plate incubated for 1.5 h, at 37 °C, under mechanical agitation at 75 rpm.

After the adhesion phase, the non-adhered cell suspension was removed by washing the wells with 150 µL of PBS twice. Subsequently, 100 µL of YNB medium containing 50 mM glucose was added to each well and the plate incubated for 48 h at 37 °C, 75 rpm. All assays were performed in quintuplicate. As a negative control, eight wells were treated identically, excluding cell contents. Biofilm quantification was performed using crystal violet staining. The medium was removed after the incubation period, and each well was washed twice with 150 µL of PBS. After removing the PBS, the plate was kept for 45 min at RT for drying. Subsequently, 110 µL of 0.4% crystal violet aqueous solution (Sigma Chemical Corporation, St Louis, MO, USA) was added to each well, followed by an incubation period of 45 min. Next, the wells were washed thrice with 300 µL of sterile milli-Q water and 200 µL of ethanol (Absolute Ethyl Alcohol P.A., Vetec, Rio de Janeiro, Brazil) 95% was added to each well and the plates incubated for another 45 min. Then, 100 µL of the decolorizing solution was transferred to a clean microtiter plate and further quantified using a spectrophotometric microplate reader (Epoch™, BioTek Instruments, Winooski, VT, USA) with a 570 nm filter. To normalize the results, the absorbance values of the negative controls were subtracted from the test samples.

The isolates were classified according to the criteria proposed by Stepanovic et al. [44] as follows: O.D._570nm_ ≤ 0.42, weak biofilm producers; 0.43 ≤ O.D._570nm_ ≤ 0.84, moderate biofilm producers; and O.D._570nm_ ≥ 0.85, strong biofilm producers.

### 2.9. Trichosporon spp. Cell Surface Hydrophobicity (CSH)

For *Trichosporon* spp. determination of the cell surface hydrophobicity (CSH), the method of Muadcheingka and Tantivitayakul was used with some modifications [45]. The yeast cells were cultivated overnight in Sabouraud broth (HiMedia, Mumbai, India) at 30 °C, 200 rpm. Then, they were washed in PBS at 2500 rpm, for 5 min, at RT. The absorbance was adjusted to an optical density (O.D._600nm_) of 1. Two milliliters of cell suspension was transferred to two glass test tubes per isolate—conducted in triplicate (the test and control tubes)—and 400 µL of hexane (Dinamica Quimica, São Paulo, Brazil) was added only to the test tubes. The tubes were incubated in a water bath (TE-056 Mag, Tecnal, Sao Paulo, Brazil) at 37 °C for 10 min, vortexed for 30 s, and returned to the water bath for another 30 min to allow for the hexane–aqueous-phase separation. The aqueous phase was carefully aspirated and transferred to acrylic cuvettes. The absorbance of both the test and control tubes was measured at 520 nm after vortexing them for 5 s. The CSH was determined with the following equation: [O.D._520nm_ (control)−O.D._520nm_ (test)/O.D._520nm_ (control)] × 100 [45].

### 2.10. Trichosporon spp. Hemolytic Activity

To evaluate the production of hemolysins, we used the methodology proposed by Luo et al. [46], with some adaptations. *Trichosporon* cells were grown in NGY medium and the inoculum standardized to 2 × 10^5^ cells/mL. An aliquot of 10 µL was taken from each standardized inoculum and spotted on the surface of SDA containing 7% fresh sheep blood (Ebe-farma) and 3% glucose, performed in triplicate. The plates were incubated for 48 h at 37 °C in 5% CO_2_ incubators. The diameter of the colonies and zones of hemolysis were measured in order to obtain the hemolysis index (HI) for each strain. The HI was determined by dividing the colony diameter (cm) by the hemolysis zone plus colony diameter (cm) measurements.

### 2.11. Trichosporon spp. Phospholipase Activity

To determine phospholipase activity, cell cultures grown overnight in NGY were standardized to a concentration of 2 × 10^5^ cells/mL and inoculated on the surface of phospholipase agar in triplicate (peptone 10 g/L; dextrose 40 g/L; Becton agar 16 g/L; egg yolk 80 g/L; NaCl 58.45 g/L; CaCl_2_ 0.55 g/L). The plates were incubated at 30 °C for 72 h. After the incubation period, the diameters of the colonies and precipitation zones were measured. The Pz (phospholipase zone) was determined by dividing the colony diameter (cm) by the precipitation zone (cm) plus colony diameter (cm) measurements [47].

### 2.12. Trichosporon spp. DNAse Activity

For the determination of DNAse activity, *Trichosporon* spp. cells were initially grown on SDA for 48 h. After the incubation period, a single colony was streaked out on the surface of DNAse agar (Kasvi, Parana, Brazil) and the Petri dishes incubated at 37 °C for 7 days. To reveal a degradation halo (DNAse activity), the plates were flooded with 10 mL of 1 M HCl [16].

### 2.13. Trichosporon spp. Cell-Wall and Plasma-Membrane Damage in the Presence of Cellular Stressors

*Trichosporon* spp. cells were cultured overnight in 5 mL of NGY broth, at 30 °C and 200 rpm. After the incubation period, cells were centrifuged at 2500 rpm for 5 min. The NGY medium was removed by washing the cells with 5 mL of PBS under the same centrifugation conditions. The pellet was resuspended in PBS and cells standardized to a concentration of 2 × 10^5^ cells/mL. Subsequently, 10-fold serial dilutions were performed and 5 µL spots were inoculated on the surface of the following culture media—YPD agar (control), YPD agar containing Congo Red (Sigma-Aldrich, Taufkirchen, Germany; 175 µg/mL), YPD agar containing Calcofluor White (Sigma-Aldrich, Germany; 28 µg/mL), and YPD agar containing Sodium Dodecyl Sulphate (SDS; Sigma-Aldrich, Germany; 12 µg/mL)—in 90 mm × 15 mm Petri dishes. The plates were incubated at 30 °C for 48 h to evaluate fungal growth [48].

### 2.14. Trichosporon spp. Antifungal Susceptibility Testing

Fluconazole (FLU), ketoconazole (KTC), itraconazole (ITC), and amphotericin B (AMB) solutions were prepared in accordance with the Clinical and Laboratory Standards Institute (CLSI) M27-A3 guidelines [49] by being diluted in RPMI 1640 medium (Roswell Park Memorial Institute; Niagara Falls, NY, USA) buffered with 3-(N-morpholino) propanesulfonic acid (MOPS) at pH 7.0. The antifungal drugs tested were serially diluted in 10 different concentrations as follows: FLU (Pfizer Incorporated, New York, NY, USA) 0.125–64 μg/mL; KTC, ITC (Pfizer Incorporated, New York, NY, USA), and AMB (Sigma Chemical Corporation, St. Louis, MO, USA) from 0.0313 to 16 μg/mL. The inocula of all strains tested were obtained from 48-h cultivation in Sabouraud broth at 30 °C and an initial cellular suspension in saline solution equivalent to the 0.5 McFarland standard was determined spectrophotometrically at 530 nm. Then, two serial dilutions were made—the first one in saline solution (1:100) and the second one in RPMI (1:20)—in order to obtain a final concentration of 10^3^ cells/mL. Aliquots of 100 µL of the final inoculum solution were dispensed in microtiter plates of 96 wells containing 100 µL of various concentrations of the drugs tested. Finally, the plates were incubated at 37 °C and test readings taken after 48-h incubation. All the strains were tested in duplicate. The MIC was defined for the azoles as the lowest drug concentration that showed an approximately 50% reduction in turbidity compared to the positive control well. For AMB, the MIC was defined as the lowest concentration able to inhibit any growth that is visually perceptible [50].

### 2.15. Statistical Analysis

Data were analyzed using the statistical software GraphPad (version 8.0). Results were presented as mean ± standard deviation, and differences were analyzed by the Mann–Whitney test. For all the analyses, *p* was considered as the default value of 0.05 with a confidence interval of 95%. In addition, the values obtained for some of the virulence attribute tests in vitro were classified into tertile categories as weak, moderate, or strong.

## 3. Results

### 3.1. Trichosporon spp. Phenotypic Screening and Molecular Identification

Twenty-five *Trichosporon* spp. isolates were evaluated in the present study, twenty of them obtained from white *piedra* (each one from a different patient), a single one from urine, two from blood cultures (same patient), and two from sequential cerebrospinal fluid (CSF) samples of the same patient. Macromorphological observation of cultures grown on SDA were compatible with *Trichosporon* spp. Arthroconidia, blastoconidia, pseudohyphae, and true hyphae were observed on the microscope slides. All the isolates were able to hydrolyze urea and did not ferment glucose.

To carry out the identification of *Trichosporon* spp. at the species level, *IGS1* rDNA fragments were amplified and the DNA sequences obtained were lodged in the GenBank database, available at the NCBI website (https://ncbi.nlm.nih.gov, accessed on 23 December 2024) for BLAST comparisons. The isolates were preliminarily identified as follows: 17 isolates as *T. inkin*, 4 isolates as *T. asahii*, and 2 for *T. faecale* and *T. asteroides* each.

### 3.2. Phylogenetic Analysis of Trichosporon spp. and Genotyping of Trichosporon asahii

**Figure 1:**
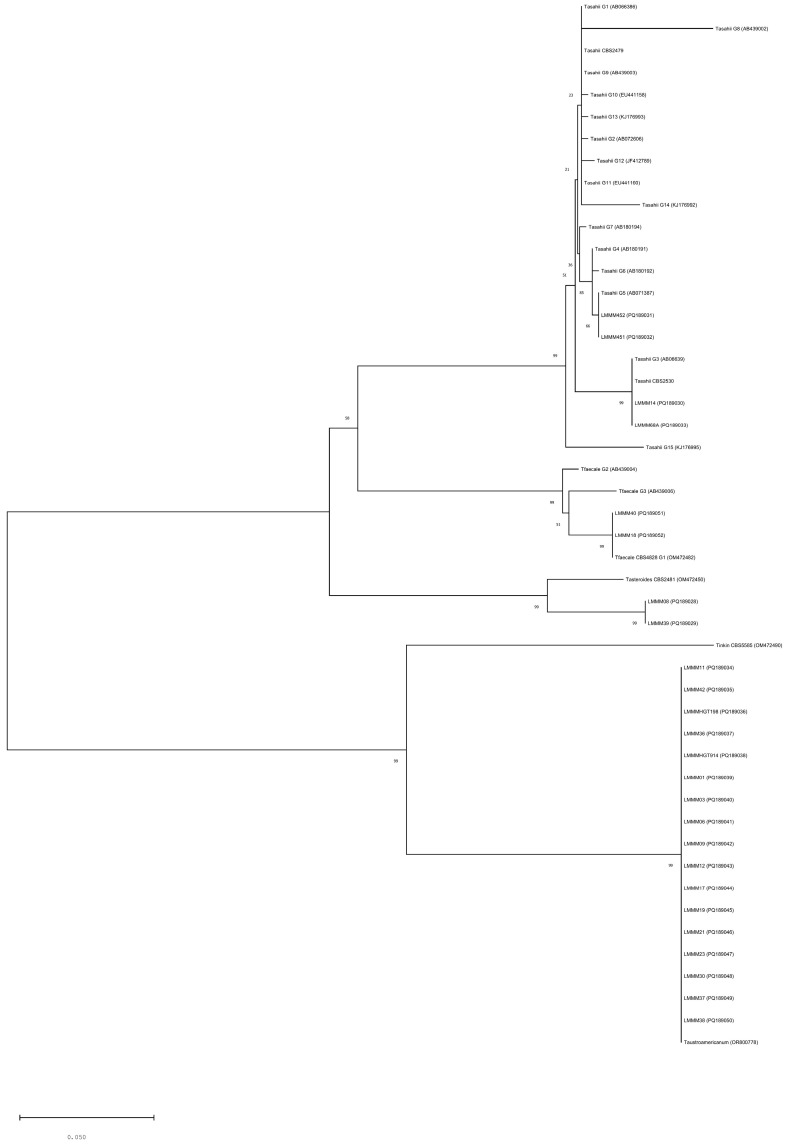
Phylogenetic analysis of 25 *Trichosporon* isolates, inferred by Neighbor-Joining method. The evolutionary distances were computed using the Kimura 2-parameter method and the percentage of replicate trees in which the associated taxa clustered together in the bootstrap test (1000 replicates) are shown next to the branches. The analysis involved 575 positions in the final dataset. Evolutionary analyses were conducted with MEGA X software. The scale bar represents the number of base substitutions per site.

Phylogenetic analysis confirmed the identification of the *Trichosporon* spp. isolates evaluated in the present study. It was also possible to observe that, in most cases, the value of the “bootstrap” (bt) was equal to 100, ensuring the accuracy of the methodology. Interestingly, the strains previously identified by BLAST actually belonged to the recently described *T. austroamericanum*, with 17 isolates. The other strains were identified as follows: *T. asahii* (n = 4), *T. faecale* (n = 2), and *T. asteroides* (n = 2). For the *T. asahii* isolates, two distinct genotypes were observed: G3 (n = 2) and G5 (n = 2). Additionally, both isolates of *T. faecale* were classified as belonging to the G1 genotype. Isolates from *T. asahii*, *T. faecale*, and *T. asteroides* were considered phylogenetically more closely related. *T. austroamericanum* isolates have been placed in another branch quite separated from the first three clades (bt = 100; Figure 1).

### 3.3. Trichosporon spp. Adhesion to Human Buccal Epithelial Cells (HBECs)

**Figure 2:**
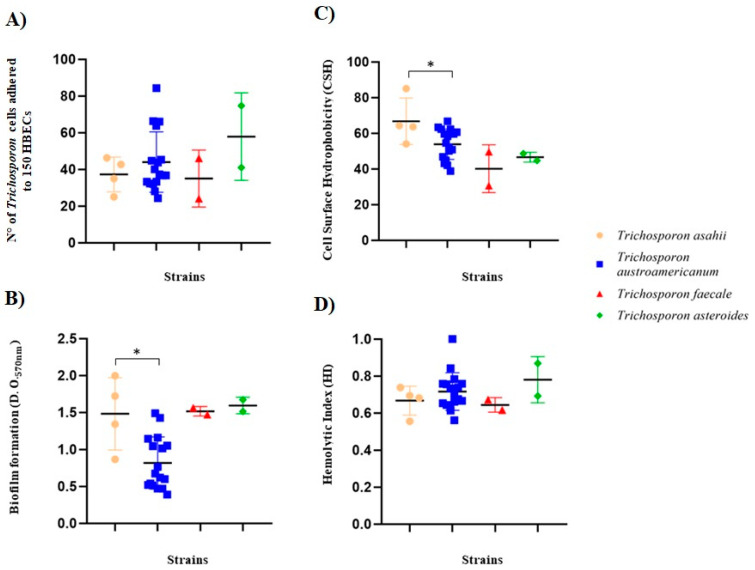
Virulence attributes of clinical strains of *Trichosporon* spp. isolated from patients in Northeast Brazil. (**A**) Adhesion to human buccal epithelial cells after 1 h incubation at a rotation of 200 rpm, 37 °C. (**B**) Biofilm formation induced after incubation of cells in 96-well microtiter plates containing YNB medium plus glucose at 37 °C for 44 h. (**C**) Cell surface hydrophobicity after incubation at 37 °C for 1 h to allow for hexane–aqueous-phase separation. (**D**) Hemolytic index after cell incubation on SDA plates—supplemented with sheep blood and glucose—at 37 °C for 48 h, 5% CO_2_. Each bar represents mean ± standard deviation obtained for each isolate. * *p* ≤ 0.05.

The 25 isolates evaluated were able to adhere to HBECs with an average of 43.3 ± 15.6 cells of *Trichosporon* spp. adhering to 150 HBECs. A great variability in the results was observed among the isolates, where the less-adherent isolate had 24 ± 0.82 *Trichosporon*/150 HBECs (LMMM18; *T. faecale*) and the highest adherent strain had 84 ± 1.25 cells of *Trichosporon*/150 HBECs (LMMM30; *T. austroamericanum*), both obtained from white *piedra* cases. *T. asahii* showed an average adhesion of 37.3 ± 9.4 cells of *Trichosporon*/150 HBECs, while *T. austroamericanum* had 44 ± 16.4 cells of *Trichosporon*/150 HBECs on average. The isolates of *T. faecale* had an average of 35 ± 15.6 *Trichosporon* cells/150 HBECs and *T. asteroides* isolates, 57.9 ± 23.8 *Trichosporon* cells/150 HBECs. The reference strain of *T. asahii* CBS2630 showed an average adhesion of 34.3 ± 1.70 *Trichosporon* cells/150 HBECs, a result within the range found in the present study. Although there was no statistically significant difference between isolates of different species, the strongest results were found among *T. austroamericanum* isolates obtained from white *piedra* (Table 1; Figure 2).

### 3.4. Trichosporon spp. Biofilm Formation

The reference strain *T. asahii* CBS2630 was categorized as a weak biofilm producer (O.D._570 nm_ of 0.40 ± 0.01), whereas most of the *Trichosporon* spp. isolates of the present study produced more biofilm than it.

All isolates of *Trichosporon* spp. were able to produce biofilm with an average absorbance (O.D._570 nm_) of 1.48 ± 0.64 for *T. asahii*, 0.82 ± 0.34 for *T. austroamericanum*, 1.52 ± 0.00 for *T. faecale*, and 1.59 ± 0.11 for *T. asteroides*. There was great variability in biofilm formation, with the O.D._570 nm_ ranging from 0.39 ± 0.01 (LMMM12; *T. austroamericanum*; white *piedra*) to 1.99 ± 0.08 (LMMM 451; *T. asahii*; blood culture).

According to Stepanovic’s classification [44], all the isolates of *T. asahii*, *T. faecale*, and *T. asteroides* were considered strong biofilm producers, whereas in *T. austroamericanum*, most isolates (58.8%) were medium-to-low biofilm producers (Table 1; Figure 2). The statistical analysis revealed that *T. asahii* strains produced greater amounts of biofilm than *T. austroamericanum* (*p* = 0.02; Figure 2).

### 3.5. Trichosporon spp. Cell Surface Hydrophobicity (CSH)

The CSH ranged from 30.6 ± 8.4 (LMMM18; *T. faecale*; white *piedra*) to 85 ± 0 (LMMM451; *T. asahii* isolate; blood culture). The reference strain *T. asahii* CBS2630 showed moderate CSH (54 ± 0.1). The same trend of higher CSH was found for *T. asahii* isolates compared to the *T. austroamericanum* counterparts (means of 66.7 ± 12.6 and 53.9 ± 8.5, respectively; *p* = 0.04). In addition, while *T. asahii* isolates were moderate (75%) or strongly (25%) hydrophobic, *T. austroamericanum* isolates were either moderate (70.6%) or weakly (29.4%) hydrophobic. All *T. faecale* and *T. asteroides* isolates showed low hydrophobicity (means of 40.1 ± 3.1 and 46.7 ± 2.8, respectively; Table 1; Figure 2).

### 3.6. Trichosporon spp. Hemolytic Activity

Only a single isolate (LMMM09; *T. austroamericanum*; white *piedra*) did not show hemolytic activity. Nevertheless, most of the isolates of all the species evaluated (56%) showed strong production of hemolysins (a low HI), with a HI ≤ 0.69, specifically among *T. asahii*, *T. faecale*, and *T. asteroides*. On the contrary, 52.9% of *T. austroamericanum* showed a moderate or low production of hemolysins, while 47% of them had a low HI (high hemolytic activity). There was no statistical difference in hemolysin production among *Trichosporon* spp. isolates. However, *T. asahii* LMMM452—obtained from blood culture—had the lowest HI (meaning the strongest hemolytic activity; 0.56 ± 0.01; Table 1; Figure 2).

### 3.7. Trichosporon spp. Phospholipase and DNAse Activity

Only two isolates obtained from white *piedra* (8%) showed very low production levels of phospholipase as follows: LMMM17 and LMMM18 with Pz values of 0.91 ± 0.08 and 0.86 ± 0, respectively (Table 1). The remaining 23 isolates (92%) did not produce the enzyme, regardless of the body site of isolation or species. It is worth mentioning that phospholipase activity is considered weak or negative when Pz values are close to 1.0. Regarding DNAse production, four *T. austroamericanum* isolates (23.5%) did not produce the enzyme. However, no remarkable differences could be observed within the different *Trichosporon* spp., where three strains (12%) were positive (+++), fourteen strains (56%) positive (++), and four strains (16%) positive (+).

### 3.8. Trichosporon spp. Cell-Wall and Membrane Stressors

**Figure 3:**
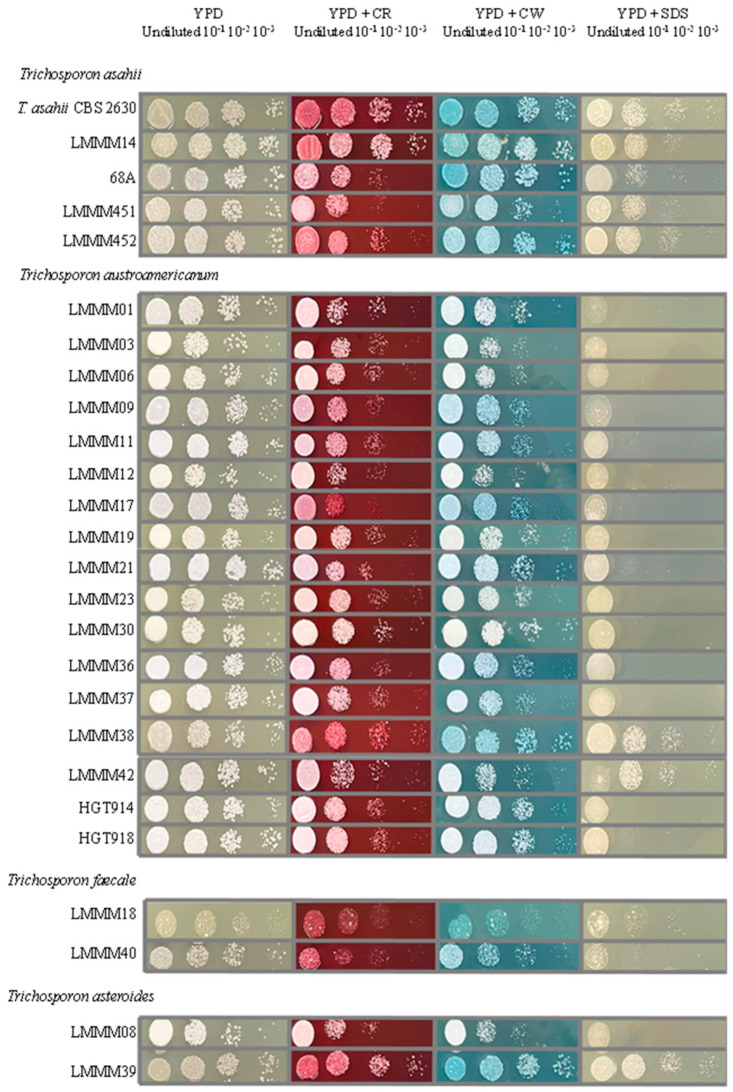
Colony growth profiling of clinical isolates of *Trichosporon* spp. in the presence of plasma-membrane and cell-wall stressors. Cell suspensions and respective dilutions were plated on YPD agar (control) and YPD agar containing the following disturbing agents: Congo Red (175 µg/mL), Calcofluor White (28 µg/mL), and SDS (12 µg/mL). The plates were incubated for 48 h at 30 °C.

It was not possible to notice a remarkable difference among *Trichosporon* spp. cells after growing in the presence of cellular disturbing agents, except for the fact that *T. asahii, T. faecale*, and *T. asteroides* strains were generally slightly more resistant to CR and CW than *T. austroamericanum*. In addition, the vast majority of the *T. austroamericanum* isolates were one or two serial dilutions more susceptible to SDS than *T. asahii* (Figure 3).

### 3.9. Trichosporon spp. Antifungal Susceptibility Testing

All MIC values obtained by the reference strains were compatible with the values expected by the CLSI methodology, guaranteeing the reliability of the results obtained for the tested isolates. MIC ranges—MIC50, MIC90, and MIC97.5 (equivalent to the epidemiological cut-off value, ECV)—and geometric means (GMs) for all the antifungal drugs tested are depicted in Table 2 once there are no breakpoints established for *Trichosporon* spp.

The FCZ MIC range obtained against all the *Trichosporon* species was from 0.5 to 8 µg/mL, with the greater values found for *T. faecale* isolates (MIC97.5 = 8; GM = 5.66). The same trend of higher MICs for this species was observed when KTC (an MIC range of 0.0313 to 0.5 µg/mL) and ITC (an MIC of 0.0313 to 1 µg/mL) were tested (MIC97.5 = 0.5 and GM = 0.25, MIC97.5 = 1 and GM = 0.5, respectively). However, the greatest MIC value was found for a strain obtained from urine (68 A) of *T. asahii* for AMB (greater than 16 µg/mL). For all the other *Trichosporon* spp., the MIC range was from 0.125 to 2 µg/mL (Table 2).

## 4. Discussion

In the present study, we identified and characterized 25 *Trichosporon* isolates obtained from white *piedra*, blood, urine, and cerebrospinal fluid. Molecular identification and phylogenetic analyses targeting the *IGS1* rDNA revealed different species of *Trichosporon* grouped into four distinct clades with well-supported branches, with *T. austroamericanum* being considered a species that is less genetically related to the others. Similar findings were reported in recent phylogeny studies conducted by Takashima et al. [51] using genomic analysis, as well as by Arastehfar et al. (2021) [52] and Takashima and Sugita [53] who revealed a greater degree of genetic relatedness among *T. asteroides, T. asahii*, and *T. faecale*. The most recent studies confirmed the previous reassignment of Tremmelomycetes performed by Liu et al. (2015) [18], who analyzed the LSU (D1/D2 domains) rDNA.

The species of *Trichosporon* identified in the present study are those considered of greater clinical relevance [3,19], together with *T. dohaense* and *T. japonicum* [52]. Most of the isolates obtained from patients with white *piedra* were identified as *T. austroamericanum* (15/20), which may corroborate with the literature because *T. inkin* (the previous species name of some *T. austroamericanum* isolates)—together with *T. ovoides* and *T. cutaneum*—are the main etiological agents of superficial infections caused by *Trichosporon* spp. [16,54]. In fact, *T. inkin* was responsible for 45% of cases of *Trichosporon* spp. superficial infections in Brazil [28]. It is mandatory that the proper identification of *T. austroamericanum* [20] is achieved in future investigations in order to check if the majority of cases of *Trichosporon* superficial infections are due to this recently described novel species rather than *T. inkin*.

In a study conducted in Mexico with 12 isolates of the genus *Trichosporon* obtained from patients with superficial infections, Martinez-Herrera et al. [55] verified that all isolates recovered from white *piedra* belonged to the species *T. inkin*. In the most recent review study with 131 cases of white *piedra*, mostly from tropical countries, *T. inkin* corresponded to 23% of cases. However, several strains were only identified at the genus level or as the no longer existing species *T. beigelii* (59.7% of the isolates). Therefore, the number of *T. inkin* isolates may be underestimated.

There were only two *T. asahii* isolates [56]. To the best of our knowledge, there are no other studies describing *T. faecale* and *T. asteroides* causing this superficial mycosis.

According to Rodriguez-Tudela et al. [57], *T. asahii* is the most prevalent *Trichosporon* species in systemic infections. Among the four isolates identified as *T. asahii*, three were obtained from urine and blood. This finding corroborates with a Brazilian study, which evaluated 358 clinical isolates of *Trichosporon* spp. where this was the most prevalent species obtained from body fluids such as urine and blood (98% and 78%, respectively) [28].

Two different genotypes were found among the four isolates identified as *T. asahii*: two belonging to G3, obtained from white *piedra* and urine, while the other two were G5, recovered from blood culture samples. By analyzing the intraspecific diversity of 284 isolates of *T. asahii* obtained from 24 medical centers in Brazil and several anatomical sources, Francisco et al. [40] reported that 7% of all *T. asahii* isolates belonged to G5. In a study conducted in Northern Brazil with the medically important species of *Trichosporon*, *Apiotrichum*, and *Cutaneotrichosporon*, Santo et al. [58] reported *T. asahii* isolates of G3 and G5 from cases of superficial infections.

The ability to adhere to epithelial and endothelial cells is described as the initial step to establishing infection among microbes [59]. All *Trichosporon* spp. isolates were able to adhere to HBECs. The *T. austroamericanum* isolates obtained from white *piedra* were medium to highly adherent to epithelial cells, which could possibly be partially explained by their need to persist on the hair shaft to cause white *piedra*. Although the buccal epithelia do not contain keratin—like human hair—*T. austroamericanum* adhesins may also more efficiently bind to HBECs. This finding could possibly explain the fact that *T. inkin* is the main species found for superficial infections.

Biofilm formation has become important for clinical practice in the field of medical mycology due to its ability to increase mortality in patients with systemic infections by *Candida* species [60]. Studies on biofilm formation in *Trichosporon* spp. have increased in recent years as this genus is considered the second-most-prevalent yeast (after *Candida* spp.) in cases of systemic infections in patients with hematological malignancies [61]. Several investigations have reported greater MICs among *Trichosporon* spp. cells that compose biofilms, rather than in planktonic-cell counterparts [62,63]. Adhesion of *T. asahii* cells to the synthetic surfaces of medical devices or host cells is the most critical phase for biofilm formation [64].

Little is known about the molecular mechanisms of biofilm formation by *Trichosporon* spp. However, Kurakado et al. [65] investigated the role of morphological transition on biofilm formation with *T. asahii* clinical isolates. They have shown that arthroconidia play a key role in biofilm formation on polystyrene substrate by *T. asahii.* Furthermore, they observed that strong biofilm-producing strains had abundant arthroconidia and showed greater CSH, corroborating the findings of the present study. In addition, the *PLA2* gene—which encodes a phospholipase belonging to an esterase superfamily—seems to be important for biofilm formation since its overexpression enhances this attribute of virulence in a mechanism that seems to be related to triggering arthroconidia production and longer true hyphae [66].

Here, we have demonstrated that *T. asahii* and their closely related species showed a strong ability to form biofilm compared to *T. austroamericanum*. However, there is a limitation in our study because the number of *T. asahii* is lower than for the *T. austroamericanum* strains. In the literature, this ability has been cited to vary among different *Trichosporon* spp. and seems to be more strain-related [13,67]. Unfortunately, most of the time, the authors do not specify the origin of the anatomical sources of isolation (superficial versus systemic), making it difficult to compare the results among them.

Another important virulence factor that was significantly higher in *T. asahii* strains was CSH. The fungal cell wall—the outermost layer of the fungal cell—maintains cell morphology, protects the cell, and transmits external stimuli to the cell cytoplasm [68]. Elevated CSH is generally considered a virulence factor in *Candida albicans* since the hydrophobic cells may influence adherence to host epithelium, germ tube formation, and decrease PMN killing [69]. In *Candida* spp., a high CSH is a common feature among strains able to cause infections [70,71]. High CSH and biofilm formation by *T. asahii* may partially explain the reason why this is the most virulent—and more frequently associated with systemic infections—*Trichosporon* species [62,72].

In the present study, *T. asahii* had significantly higher CSH and were considered strong biofilm producers. The same trends of strong-biofilm-producing strains also presenting high CSH were recently described by Kurakado et al. (2021) [65] using a water–hydrocarbon two-phase assay, the same methodology used in the present study. Conversely, Ichikawa et al. [73] did not find a positive correlation between these two virulence factors in *T. asahii*. This finding may be explained by the methodology adopted by the authors because the CSH was assessed with an assay based on adhesion to polystyrene microspheres.

Some dyes such as CR and CW have been used as fungal cell-wall stressors. Both compounds have two sulfonic acid groups, which are negatively charged under slightly acidic to basic conditions [74]. In fungi, CR binds to β-1,3-glucans, whereas CW has a higher affinity to chitin [75]. CR affects the transcription of genes related to primary and secondary metabolism and toxin efflux systems, suggesting that damage to the cell wall may cause serious adverse effects on fungal growth [76]. On the other hand, SDS is an anionic surfactant used in studies of cell-plasma-membrane damage but it also affects cell wall integrity [1]. It seems that *T. austroamericanum* is more sensitive to cell stressors, specifically SDS. This finding should be better investigated with a higher number of strains. It is possible that the greater resistance of *T. asahii* to SDS is linked to its higher MICs against AMB since the genes related to ergosterol metabolism (*ERG2*, *ERG3*, and *ERG4*) are overexpressed in a *Saccharomyces cerevisiae* strain resistant to SDS [1].

Hemolysins are pore-forming toxins able to lyse erythrocytes by destroying the heme factor to release iron, an essential component for several fungal species’ growth [46]. Sun et al. [14] demonstrated variable HI in *T. asahii* isolates obtained from urine samples belonging to the G1, G3, and G5 genotypes. There was only one white *piedra* sample that did not produce this enzyme, corroborating our findings. Here, the ability to express this virulence factor was not related to the different *Trichosporon* species or body sites. Hemolysin production in *T. asahii* seems to be controversial. While some authors report the expression of this virulent factor in vitro for most strains of different genotypes, Montoya et al. [16] were unable to observe hemolytic activity in the urine strains, with the exception of one skin isolate of *T. asahii* that belonged to G7.

The ability to secrete extracellular phospholipases is well-understood in *Candida* species, where these enzymes degrade host cell membranes, leading to cell disruption and subsequent tissue invasion. In this yeast species, there is a correlation between the greater production of phospholipases and crude mortality in a murine model of systemic infection [77].

Among the isolates evaluated, only two strains obtained from white *piedra* showed inexpressive phospholipase activity, whereas all the other strains did not produce the enzyme, corroborating the findings of Sun et al. [14] and Montoya et al. [16], who did not observe phospholipase production for clinical isolates from different body sites. These findings may suggest that *Trichosporon* spp. have different mechanisms for obtaining fatty acids to supply their nutritional necessities or that the assay used is not the most appropriate to assess phospholipase production in *Trichosporon* spp.

DNAse activity is a virulence factor widely known for bacteria, such as those of the genus Staphylococcus [78]. In fungi, Sanchez et al. [79] observed that clinical strains of Cryptococcus spp. had greater DNAse activity compared to the environmental isolate counterparts. The authors suggest that this could be an attribute of virulence. Different levels of DNAse activity do not seem to be related to any Trichosporon-specific species because it may vary among different strains of the same species. The findings of the present study are in agreement with other investigations [16,80].

Currently, there are no clinical breakpoints for the interpretation of MICs for *Trichosporon* spp. to antifungal drugs. However, it is recommended to carry out antifungal susceptibility testing to provide data for epidemiological surveillance and to determine epidemiological cut-off values and other parameters for helping clinicians adopt proper therapy to treat trichosporonosis [81].

It is worth mentioning that the two *T. faecale* isolates (LMMM18 and LMMM40) obtained from white *piedra* had higher MICs against the azoles than the other *Trichosporon* spp. This finding reinforces the idea that every single strain should be tested for antifungal susceptibility, once topical antifungals drugs like KTC—or even oral antifungals (ITC and FLU)—are used to eliminate scalp carriage and infection [82].

On the other hand, the highest MIC against AMB was found for *T. asahii* (isolate 68 A) obtained from urine. This may be related to the fact that this strain was obtained from a hospitalized individual that could have reached the urinary tract after systemic dissemination. This is consistent with the review performed by Arastehfar et al. [52], where the highest MICs for this antifungal were in general found for this species (values up to 64 µg/mL). Recently, Francisco et al. [28] proposed the epidemiological cut-off value (ECV) for *T. asahii* AMB (4 µg/mL). Therefore, this strain could be categorized as a non-wild-type phenotype.

## 5. Conclusions

It is possible to conclude that *Trichosporon* spp. may present peculiarities in terms of expression of different virulence factors in vitro, besides displaying a variable susceptibility to different cellular stressors. There is a trend for higher biofilm formation and CSH in *T. asahii*. In addition, the most virulent *Trichosporon* species also seems to be more resistant to cell-wall and plasma-membrane disturbing agents. On the other hand, *T. austroamericanum* seems to effectively adhere to epithelial cells. *T. faecale* isolates may present high MICs to the azoles, while *T. asahii* against amphotericin B.

## Figures and Tables

**Table 1:** Virulence factors of *Trichosporon* spp. clinical isolates from patients with superficial and disseminated trichosporonosis in Natal, Rio Grande do Norte State, Brazil. Dark-gray dashed borders stand for strong production (+++), whereas light gray means moderate production (++). Non-colored values mean weak or negative expressions of virulence factors (+).

Strain Name	Source	Nº of *Trichosporon* Cells Adhered to 150 HBECs	Biofilm Formation (D.O._570nm_)	Cell Surface Hydrophobicity (CSH)	Hemolytic Index (HI)	Phospholipase Zone (Pz)	DNAse
***Trichosporon asahii***							
CBS2630		34.3 ± 1.7	0.40 ± 0.01	54.5 ± 0.1	0.74 ± 0.01	1 ± 0	
LMMM14	White *Piedra*	35 ± 0.82	0.87 ± 0.01	54.05 ± 0.65	0.68 ± 0.02	1 ± 0	(++)
68A	Urine	25 ± 2	1.34 ± 0.04	64.3 ± 0.4	0.74 ± 0.01	1 ± 0	(++)
LMMM451	Blood	42.7 ± 2.08	1.99 ± 0.08	85 ± 0	0.69 ± 0.02	1 ± 0	(++)
LMMM452	Blood	46.3 ± 3.61	1.72 ± 0.06	63.6 ± 0	0.56 ± 0.01	1 ± 0	(+++)
***Trichosporon austroamericanum***							
LMMM01	White *Piedra*	44.7 ± 0.47	1.16 ± 0.05	46.75 ± 4.65	0.84 ± 0.01	1 ± 0	Negative
LMMM03	White *Piedra*	28 ± 0.82	1.05 ± 0.08	43.15 ± 2.15	0.74 ± 0.01	1 ± 0	(+)
LMMM06	White *Piedra*	36.7 ± 0.47	1.14 ± 0.06	60.65 ± 2.15	0.66 ± 0.01	1 ± 0	(++)
LMMM09	White *Piedra*	63.7 ± 0.47	1.02 ± 0.06	54.5 ± 0	1 ± 0	1 ± 0	(++)
LMMM11	White *Piedra*	66 ± 3.56	0.51 ± 0.01	62.3 ± 0.1	0.76 ± 0.01	1 ± 0	(++)
LMMM12	White *Piedra*	43.7 ± 0.94	0.39 ± 0.01	66.7 ± 0.1	0.65 ± 0.02	1 ± 0	(++)
LMMM17	White *Piedra*	45.3 ± 1.25	0.62 ± 0.02	38.8 ± 0.4	0.63 ± 0.04	0.91 ± 0.08	(+)
LMMM19	White *Piedra*	33.3 ± 1.25	0.54 ± 0.03	42.05 ± 2.95	0.76 ± 0.04	1 ± 0	(+++)
LMMM21	White *Piedra*	24.3 ± 0.47	1.49 ± 0.01	52.1 ± 0.3	0.76 ± 0.09	1 ± 0	(++)
LMMM23	White *Piedra*	32.3 ± 1.25	0.47 ± 0.03	59.6 ± 2.2	0.61 ± 0.02	1 ± 0	(++)
LMMM30	White *Piedra*	84.3 ± 1.25	0.52 ± 0.02	50.75 ± 3.55	0.73 ± 0.02	1 ± 0	(++)
LMMM36	White *Piedra*	33.3 ± 1.7	1.43 ± 0.02	63.35 ± 3.85	0.67 ± 0.04	1 ± 0	(++)
LMMM37	White *Piedra*	40 ± 1.63	0.60 ± 0.01	62.25 ± 2.15	0.78 ± 0.03	1 ± 0	(++)
LMMM38	White *Piedra*	32 ± 1.63	0.67 ± 0.02	59.6 ± 0.2	0.64 ± 0.01	1 ± 0	Negative
LMMM42	White *Piedra*	66.3 ± 2.49	0.47 ± 0.03	50.15 ± 2.15	0.70 ± 0.01	1 ± 0	(++)
HGT914	Liquor	37.3 ± 1.53	1.05 ± 0.01	58.25 ± 0.15	0.56 ± 0.01	1 ± 0	Negative
HGT198	Liquor	36.7 ± 1.53	0.77 ± 0.05	44.9 ± 0.5	0.68 ± 0.01	1 ± 0	Negative
***Trichosporon faecale***							
LMMM18	White *Piedra*	24 ± 0.82	1.56 ± 0.03	30.65 ± 8.45	0.67 ± 0.05	0.86 ± 0	(++)
LMMM40	White *Piedra*	46 ± 0.82	1.47 ± 0.03	49.6 ± 4	0.62 ± 0.01	1 ± 0	(+++)
***Trichosporon asteroides***							
LMMM08	White *Piedra*	41 ± 0.82	1.51 ± 0.05	48.6 ± 3.2	0.69 ± 0.01	1 ± 0	(+)
LMMM39	White *Piedra*	74.7 ± 1.7	1.67 ± 0.09	44.7 ± 0.1	0.87 ± 0.01	1 ± 0	(+)

**Table 2:** Pooled MIC distribution, geometric means, MIC range—MIC50, MIC90, and MIC97.5—of 25 clinical isolates of *Trichosporon* spp. obtained from patients diagnosed with trichosporonosis in Rio Grande do Norte, Northeast Brazil, as determined by the CLSI broth microdilution method.

Antifungal Drug	Species	MIC 50	MIC 90	MIC 97.5	MIC Geometric Mean (μg/mL)	Nº of Isolates Tested	Nº of Isolates with an MIC (μg/mL) of									
							0.0313	0.0625	0.125	0.25	0.5	1	2	4	8	>16
**Fluconazole**	***Trichosporon asahii***	1	2	2	1.41	4						2	2			
	***Trichosporon austroamericanum***	2	2	2	1.39	17					1	7	9			
	***Trichosporon faecale***	4	8	8	5.66	2								1	1	
	***Trichosporon asteroides***	0.5	2	2	1	2					1		1			
**Ketoconazole**	***Trichosporon asahii***	0.0313	0.25	0.25	0.06	4	2	1		1						
	***Trichosporon austroamericanum***	0.0625	0.125	0.125	0.05	17	7	7	3							
	***Trichosporon faecale***	0.125	0.5	0.5	0.25	2			1		1					
	***Trichosporon asteroides***	0.0313	0.0313	0.0313	0.03	2	2									
**Itraconazole**	***Trichosporon asahii***	0.125	0.125	0.125	0.11	4		1	3							
	***Trichosporon austroamericanum***	0.0625	0.25	0.25	0.08	17	3	7	6	1						
	***Trichosporon faecale***	0.25	1	1	0.5	2				1		1				
	***Trichosporon asteroides***	0.0313	0.125	0.125	0.06	2	1		1							
**Amphotericin B**	***Trichosporon asahii***	0.125	>16	>16	0.25	4			2			1				1
	***Trichosporon austroamericanum***	0.5	2	2	2	17			1	2	8	5	1			
	***Trichosporon faecale***	0.5	2	2	1	2					1		1			
	***Trichosporon asteroides***	0.25	1	1	0.5	2				1		1				

Note: MIC = minimum inhibitory concentration.

## Data Availability

The original contributions presented in this study are included in the article. Further inquiries can be directed to the corresponding author.

## References

[1] Matsumoto Y., Yoshikawa A., Nagamachi T., Sugiyama Y., Yamada T., Sugita T. (2022). A Critical Role of Calcineurin in Stress Responses, Hyphal Formation, and Virulence of the Pathogenic Fungus *Trichosporon asahii*. Sci. Rep..

[2] Lara B.R., De Camargo B.B., Paula C.R., Junior D.P.L., Garces H.G., Arnoni M.V., Silveira M., Gimenes V.M.F., Siqueira L.P.M.H., Takahashi J.P.F. (2021). Comparing the Phenotypic, Genotypic, and Proteomic Identification of *Trichosporon* Species: A Globally Emerging Yeast of Medical Importance. Med. Mycol..

[3] de Andrade I.B., de Sousa Araújo G.R., Brito-Santos F., Figueiredo-Carvalho M.H.G., Zancopé-Oliveira R.M., Frases S., Almeida-Paes R. (2022). Comparative Biophysical and Ultrastructural Analysis of Melanins Produced by Clinical Strains of Different Species From the Trichosporonaceae Family. Front. Microbiol..

[4] Colombo A.L., Padovan A.C.B., Chaves G.M. (2011). Current Knowledge of *Trichosporon* Spp. and Trichosporonosis. Clin. Microbiol. Rev..

[5] Mariné M., Brown N.A., Riaño-Pachón D.M., Goldman G.H. (2015). On and Under the Skin: Emerging Basidiomycetous Yeast Infections Caused by *Trichosporon* Species. PLoS Pathog..

[6] Parashar A., Rastogi V., Rudramurthy S.M., Ghosh A.K., Chander J., Kindo A.J. (2022). Faster and Accurate Identification of Clinically Important *Trichosporon* Using MALDI TOF MS. Indian J. Med. Microbiol..

[7] Chagas-Neto T.C., Chaves G.M., Colombo A.L. (2008). Update on the Genus *Trichosporon*. Mycopathologia.

[8] de Magalhães A.R., Nishikawa M.M., de Mondino S.S.B., de Macedo H.W., da Silva da Rocha E.M., de Souza Baptista A.R. (2016). *Trichosporon* Isolation from Human Ungueal Infections: Is There a Pathogenic Role?. An. Bras. Dermatol..

[9] Inácio C.P., Rocha A.P.S., Barbosa R.d.N., Oliveira N.T., Silva J.C., de Lima-Neto R.G., Macêdo D.P.C., Neves R.P. (2016). Experimental White Piedra: A Robust Approach to Ultrastructural Analysis, Scanning Electron Microscopy and Etiological Discoveries. Exp. Dermatol..

[10] de Carvalho Ribeiro C.S., Zaitz C., de Souza Framil V.M., de Carvalho Ottoboni T.S., de Carvalho Tonoli M.S., Ribeiro R.P. (2015). Descriptive Study of Onychomycosis in a Hospital in São Paulo. Braz. J. Microbiol..

[11] Milan E.P., Silva-Rocha W.P., De Almeida J.J.S., Fernandes T.U.G., De Araújo Prudente A.L., De Azevedo M.F., Francisco E.C., De Azevedo Melo A.S., Colombo A.L., Chaves G.M. (2018). *Trichosporon inkin* Meningitis in Northeast Brazil: First Case Report and Review of the Literature. BMC Infect. Dis..

[12] da Silva Pontes Z.B.V., Ramos A.L., de Oliveira Lima E., de Fátima de Lacerda Guerra M., Oliveira N.M.C., dos Santos J.P. (2002). Clinical and Mycological Study of Scalp White Piedra in the State of Paraíba, Brazil. Mem. Inst. Oswaldo Cruz.

[13] Iturrieta-González I.A., Padovan A.C.B., Bizerra F.C., Hahn R.C., Colombo A.L. (2014). Multiple Species of *Trichosporon* Produce Biofilms Highly Resistant to Triazoles and Amphotericin B. PLoS ONE.

[14] Sun W., Su J., Xu S., Yan D. (2012). *Trichosporon asahii* Causing Nosocomial Urinary Tract Infections in Intensive Care Unit Patients: Genotypes, Virulence Factors and Antifungal Susceptibility Testing. J. Med. Microbiol..

[15] Pfaller M.A., Diekema D.J. (2004). Rare and Emerging Opportunistic Fungal Pathogens: Concern for Resistance beyond *Candida albicans* and *Aspergillus fumigatus*. J. Clin. Microbiol..

[16] Montoya A.M., González A.S., Palma-Nicolás J.P., Gómez-Treviño A., González J.G., González G.M. (2015). Genotyping, Extracellular Compounds, and Antifungal Susceptibility Testing of *Trichosporon asahii* Isolated from Mexican Patients. Med. Mycol..

[17] Rastogi V., Honnavar P., Rudramurthy S.M., Pamidi U., Ghosh A., Chakrabarti A. (2016). Molecular Characterisation and Antifungal Susceptibility of Clinical *Trichosporon* Isolates in India. Mycoses.

[18] Liu X.Z., Wang Q.M., Göker M., Groenewald M., Kachalkin A.V., Lumbsch H.T., Millanes A.M., Wedin M., Yurkov A.M., Boekhout T. (2015). Towards an Integrated Phylogenetic Classification of the *Tremellomycetes*. Stud. Mycol..

[19] Wang L., Wang Q.M. (2015). Molecular Phylogenetic Analysis of Ballistoconidium-Forming Yeasts in Trichosporonales (*Tremellomycetes*): A Proposal for *Takashimella* gen. nov. and *Cryptotrichosporon tibetense* sp. nov. PLoS ONE.

[20] Francisco E.C., Desnos-Ollivier M., Dieleman C., Boekhout T., Santos D.W.d.C.L., Medina-Pestana J.O., Colombo A.L., Hagen F. (2024). Unveiling *Trichosporon austroamericanum* sp. nov.: A Novel Emerging Opportunistic Basidiomycetous Yeast Species. Mycopathologia.

[21] Caggiano G., Iatta R., Laneve A., Manca F., Montagna M.T. (2008). Observational Study on Candidaemia at a University Hospital in Southern Italy from 1998 to 2004. Mycoses.

[22] Fonseca F.L., Frases S., Casadevall A., Fischman-Gompertz O., Nimrichter L., Rodrigues M.L. (2009). Structural and Functional Properties of the *Trichosporon asahii* Glucuronoxylomannan. Fungal Genet. Biol..

[23] Mehta V., Nayyar C., Gulati N., Singla N., Rai S., Chandar J. (2021). A Comprehensive Review of *Trichosporon* spp.: An Invasive and Emerging Fungus. Cureus.

[24] Caira M., Trecarichi E.M., Tumbarello M., Leone G., Pagano L. (2011). Uncommon Yeast Infections in Hematological Patients: From Diagnosis to Treatment. Expert Rev. Anti-Infect. Ther..

[25] Rodriguez-Tudela J.L., Diaz-Guerra T.M., Mellado E., Cano V., Tapia C., Perkins A., Gomez-Lopez A., Rodero L., Cuenca-Estrella M. (2005). Susceptibility Patterns and Molecular Identification of *Trichosporon* Species. Antimicrob. Agents Chemother..

[26] Messias A., Junior S., Bandeira M.A., Miranda R., de Camargo Z.P. (2010). *Trichosporon* Species Isolated from the Perigenital Region, Urine and Catheters of a Brazilian Population. Braz. J. Microbiol..

[27] Diaz M.R., Fell J.W. (2004). High-Throughput Detection of Pathogenic Yeasts of the Genus *Trichosporon*. J. Clin. Microbiol..

[28] Francisco E.C., de Almeida Junior J.N., de Queiroz Telles F., Aquino V.R., Mendes A.V.A., de Andrade Barberino M.G.M., de Tarso P., Guimarães T., Hahn R.C., Padovan A.C.B. (2019). Species Distribution and Antifungal Susceptibility of 358 *Trichosporon* Clinical Isolates Collected in 24 Medical Centres. Clin. Microbiol. Infect..

[29] Guo L.N., Yu S.Y., Hsueh P.R., Al-Hatmi A.M.S., Meis J.F., Hagen F., Xiao M., Wang H., Barresi C., Zhou M.L. (2019). Invasive Infections Due to *Trichosporon*: Species Distribution, Genotyping, and Antifungal Susceptibilities from a Multicenter Study in China. J. Clin. Microbiol..

[30] Matsue K., Uryu H., Koseki M., Asada N., Takeuchi M. (2006). Breakthrough Trichosporonosis in Patients with Hematologic Malignancies Receiving Micafungin. Clin. Infect. Dis..

[31] Fischman O., Bezerra F.C., Francisco E.C., da Silva F.C., Nishikaku A.S., Cavalcanti S.D.B., de Azevedo Melo A.S., Bentubo H.D.L., Petri V. (2014). *Trichosporon inkin*: An Uncommon Agent of Scalp White Piedra. Report of Four Cases in Brazilian Children. Mycopathologia.

[32] Silva-Rocha W.P., de Azevedo M.F., Chaves G.M. (2017). Épidémiologie et Distribution Des Espèces Fongiques Des Mycoses Superficielles Dans Le Nord-Est Du Brésil. J. Mycol. Med..

[33] Sugita T., Nakajima M., Ikeda R., Matsushima T., Shinoda T. (2002). Sequence Analysis of the Ribosomal DNA Intergenic Spacer 1 Regions of *Trichosporon* Species. J. Clin. Microbiol..

[34] Gordon D. (2003). Viewing and Editing Assembled Sequences Using Consed. Curr. Protoc. Bioinform..

[35] Ewing B., Green P. (1998). Base-Calling of Automated Sequencer Traces Using Phred. II. Error Probabilities. Genome Res..

[36] Kumar S., Stecher G., Li M., Knyaz C., Tamura K. (2018). MEGA X: Molecular Evolutionary Genetics Analysis across Computing Platforms. Mol. Biol. Evol..

[37] Felsenstein J. (1985). Confidence Limits on Phylogenies: An Approach Using the Bootstrap. Evolution.

[38] Kimura M. (1980). A Simple Method for Estimating Evolutionary Rates of Base Substitutions through Comparative Studies of Nucleotide Sequences. J. Mol. Evol..

[39] Saitou N., Nei M. (1987). The Neighbor-Joining Method: A New Method for Reconstructing Phylogenetic Trees. Mol. Biol. Evol..

[40] Francisco E.C., de Almeida Junior J.N., Queiroz-Telles F., Aquino V.R., Mendes A.V.A., de Oliveira Silva M., Castro P.d.T.O.e., Guimarães T., Ponzio V., Hahn R.C. (2021). Correlation of *Trichosporon asahii* Genotypes with Anatomical Sites and Antifungal Susceptibility Profiles: Data Analyses from 284 Isolates Collected in the Last 22 Years across 24 Medical Centers. Antimicrob. Agents Chemother..

[41] Chaves G.M., Bates S., MacCallum D.M., Odds F.C. (2007). *Candida albicans* GRX2, Encoding a Putative Glutaredoxin, Is Required for Virulence in a Murine Model. Genet. Mol. Res..

[42] Bates S., MacCallum D.M., Bertram G., Munro C.A., Hughes H.B., Buurman E.T., Brown A.J.P., Odds F.C., Gow N.A.R. (2005). *Candida albicans* Pmr1p, a Secretory Pathway P-Type Ca^2+^/Mn^2+^-ATPase, Is Required for Glycosylation and Virulence. J. Biol. Chem..

[43] Jin Y., Yip H.K., Samaranayake Y.H., Yau J.Y., Samaranayake L.P. (2003). Biofilm-Forming Ability of *Candida albicans* Is Unlikely to Contribute to High Levels of Oral Yeast Carriage in Cases of Human Immunodeficiency Virus Infection. J. Clin. Microbiol..

[44] Stepanović S., Vuković D., Dakić I., Savić B., Švabić-Vlahović M. (2000). A Modified Microtiter-Plate Test for Quantification of Staphylococcal Biofilm Formation. J. Microbiol. Methods.

[45] Muadcheingka T., Tantivitayakul P. (2015). Distribution of *Candida albicans* and Non-Albicans Candida Species in Oral Candidiasis Patients: Correlation between Cell Surface Hydrophobicity and Biofilm Forming Activities. Arch. Oral Biol..

[46] Luo G., Samaranayake L.P., Yau J.Y.Y. (2001). Candida Species Exhibit Differential in Vitro Hemolytic Activities. J. Clin. Microbiol..

[47] Price M.F., Wilkinson I.D., Gentry L.O. (1982). Plate Method for Detection of Phospholipase Activity in *Candida albicans*. Med. Mycol..

[48] Gil-Bona A., Parra-Giraldo C.M., Hernáez M.L., Reales-Calderon J.A., Solis N.V., Filler S.G., Monteoliva L., Gil C. (2015). *Candida albicans* Cell Shaving Uncovers New Proteins Involved in Cell Wall Integrity, Yeast to Hypha Transition, Stress Response and Host-Pathogen Interaction. J. Proteom..

[49] (2008). Reference Method for Broth Dilution Antifungal Susceptibility Testing of Filamentous Fungi; Approved Standard—Second Edition.

[50] (2012). Reference Method for Broth Dilution Antifungal Susceptibility Testing of Yeasts; Fourth Informational Supplement.

[51] Takashima M., Manabe R.-i., Nishimura Y., Endoh R., Ohkuma M., Sriswasdi S., Sugita T., Iwasaki W. (2019). Recognition and Delineation of Yeast Genera Based on Genomic Data: Lessons from Trichosporonales. Fungal Genet. Biol..

[52] Arastehfar A., de Almeida Júnior J.N., Perlin D.S., Ilkit M., Boekhout T., Colombo A.L. (2021). Multidrug-Resistant *Trichosporon* Species: Underestimated Fungal Pathogens Posing Imminent Threats in Clinical Settings. Crit. Rev. Microbiol..

[53] Takashima M., Sugita T. (2019). Draft Genome Analysis of Trichosporonales Species That Contribute to the Taxonomy of the Genus *Trichosporon* and Related Taxa. Med. Mycol. J..

[54] Robles-Tenorio A., Lepe-Moreno K.Y., Mayorga-Rodríguez J. (2020). White Piedra, a Rare Superficial Mycosis: An Update. Curr. Fungal Infect. Rep..

[55] Martínez-Herrera E., Duarte-Escalante E., del Rocío Reyes-Montes M., Arenas R., Acosta-Altamirano G., Moreno-Coutiño G., Vite-Garín T.M., Meza-Robles A., Frías-De-León M.G. (2021). Molecular Identification of Yeasts from the Order Trichosporonales Causing Superficial Infections. Rev. Iberoam. Micol..

[56] Guerrero-Ponce A.E., Araiza J., Tirado-Sánchez A., Bonifaz A. (2024). Review Article White Piedra: Review of 131 Cases. Mycoses.

[57] Rodriguez-Tudela J.L., Gomez-Lopez A., Alastruey-Izquierdo A., Mellado E., Bernal-Martinez L., Cuenca-Estrella M. (2007). Genotype Distribution of Clinical Isolates of *Trichosporon asahii* Based on Sequencing of Intergenic Spacer 1. Diagn. Microbiol. Infect. Dis..

[58] do Espírito Santo E.P.T., Monteiro R.C., da Costa A.R.F., Marques-da-Silva S.H. (2020). Molecular Identification, Genotyping, Phenotyping, and Antifungal Susceptibilities of Medically Important *Trichosporon*, Apiotrichum, and Cutaneotrichosporon Species. Mycopathologia.

[59] Maza P.K., Bonfim-Melo A., Padovan A.C.B., Mortara R.A., Orikaza C.M., Ramos L.M.D., Moura T.R., Soriani F.M., Almeida R.S., Suzuki E. (2017). *Candida albicans*: The Ability to Invade Epithelial Cells and Survive under Oxidative Stress Is Unlinked to Hyphal Length. Front. Microbiol..

[60] Tumbarello M., Fiori B., Trecarichi E.M., Posteraro P., Losito A.R., de Luca A., Sanguinetti M., Fadda G., Cauda R., Posteraro B. (2012). Risk Factors and Outcomes of Candidemia Caused by Biofilm-Forming Isolates in a Tertiary Care Hospital. PLoS ONE.

[61] Yang S., Liao Y., Cong L., Lu X., Yang R. (2016). In Vitro Interactions between Non-Steroidal Anti-Inflammatory Drugs and Antifungal Agents against Planktonic and Biofilm Forms of *Trichosporon asahii*. PLoS ONE.

[62] Di Bonaventura G., Pompilio A., Picciani C., Iezzi M., D’Antonio D., Piccolomini R. (2006). Biofilm Formation by the Emerging Fungal Pathogen *Trichosporon asahii*: Development, Architecture, and Antifungal Resistance. Antimicrob. Agents Chemother..

[63] Cordeiro R.d.A., Aguiar A.L.R., da Silva B.N., Pereira L.M.G., Portela F.V.M., de Camargo Z.P., de Lima-Neto R.G., Castelo-Branco D.d.S.C.M., Rocha M.F.G., Sidrim J.J.C. (2021). *Trichosporon asahii* and *Trichosporon inkin* Biofilms Produce Antifungal-Tolerant Persister Cells. Front. Cell. Infect. Microbiol..

[64] Liao Y., Zhao H., Lu X., Yang S., Zhou J., Yang R. (2015). Efficacy of Ethanol against *Trichosporon asahii* Biofilm in Vitro. Med. Mycol..

[65] Kurakado S., Miyashita T., Chiba R., Sato C., Matsumoto Y., Sugita T. (2021). Role of Arthroconidia in Biofilm Formation by *Trichosporon asahii*. Mycoses.

[66] Ma X., Liu H., Liu Z., Wang Y., Zhong Z., Peng G., Gu Y. (2023). *Trichosporon asahii* PLA2 Gene Enhances Drug Resistance to Azoles by Improving Drug Efflux and Biofilm Formation. Int. J. Mol. Sci..

[67] Lara B.R., de Camargo B.B., Paula C.R., Monari G.P.d.M., Garces H.G., Arnoni M.V., Silveira M., Gimenes V.M.F., Leite Junior D.P., Bonfietti L.X. (2023). Aspects Related to Biofilm Production and Antifungal Susceptibility of Clinically Relevant Yeasts of the Genus *Trichosporon*. Med. Mycol..

[68] Yoshimi A., Miyazawa K., Kawauchi M., Abe K. (2022). Cell Wall Integrity and Its Industrial Applications in Filamentous Fungi. J. Fungi.

[69] Hazen K.C., Lay J.-G., Hazen B.W., Fu R.C., Seetha A. (1990). Partial Biochemical Characterization of Cell Surface Hydrophobicity and Hydrophilicity of *Candida albicans*. Infect. Immun..

[70] Dabiri S., Shams-Ghahfarokhi M., Razzaghi-Abyaneh M. (2018). Comparative Analysis of Proteinase, Phospholipase, Hydrophobicity and Biofilm Forming Ability in Candida Species Isolated from Clinical Specimens. J. Mycol. Med..

[71] Galán-Ladero M.A., Blanco-Blanco M.T., Hurtado C., Pérez-Giraldo C., Blanco M.T., Gómez-García A.C. (2013). Determination of Biofilm Production by Candida Tropicalis Isolated from Hospitalized Patients and Its Relation to Cellular Surface Hydrophobicity, Plastic Adherence and Filamentation Ability. Yeast.

[72] de Andrade I.B., Figueiredo-Carvalho M.H.G., Chaves A.L.d.S., Coelho R.A., Almeida-Silva F., Zancopé-Oliveira R.M., Frases S., Brito-Santos F., Almeida-Paes R. (2023). Metabolic and Phenotypic Plasticity May Contribute for the Higher Virulence of *Trichosporon asahii* over Other Trichosporonaceae Members. Mycoses.

[73] Ichikawa T., Hirata C., Takei M., Tagami N., Murasawa H., Ikeda R. (2017). Cell Surface Hydrophobicity and Colony Morphology of *Trichosporon asahii* Clinical Isolates. Yeast.

[74] Ram A.F.J., Klis F.M. (2006). Identification of Fungal Cell Wall Mutants Using Susceptibility Assays Based on Calcofluor White and Congo Red. Nat. Protoc..

[75] Wood P.J. (1980). Specificity in the interaction of direct dys with polysaccharides. Carbohydr. Res..

[76] Liu Z., Raj S., van Rhijn N., Fraczek M., Michel J.P., Sismeiro O., Legendre R., Varet H., Fontaine T., Bromley M. (2021). Functional Genomic and Biochemical Analysis Reveals Pleiotropic Effect of Congo Red on *Aspergillus fumigatus*. MBio.

[77] Ellepola A.N.B., Samaranayake L.P., Khan Z.U. (2016). Extracellular Phospholipase Production of Oral *Candida albicans* Isolates from Smokers, Diabetics, Asthmatics, Denture Wearers and Healthy Individuals Following Brief Exposure to Polyene, Echinocandin and Azole Antimycotics. Braz. J. Microbiol..

[78] Kateete D.P., Kimani C.N., Katabazi F.A., Okeng A., Okee M.S., Nanteza A., Joloba M.L., Najjuka F.C. (2010). Identification of Staphylococcus Aureus: DNase and Mannitol Salt Agar Improve the Efficiency of the Tube Coagulase Test. Ann. Clin. Microbiol. Antimicrob..

[79] Sánchez M., Colom F. (2010). Extracellular DNase Activity of *Cryptococcus neoformans* and *Cryptococcus gattii*. Rev. Iberoam. Micol..

[80] Bentubo H.D.L., Gompertz O.F. (2014). Effects of Temperature and Incubation Time on the in vitro Expression of Proteases, Phospholipases, Lipases and DNases by Different Species of *Trichosporon*. Springerplus.

[81] Sprute R., Bethe U., Chen S.C.A., Cornely O.A. (2022). EQUAL *Trichosporon* Score 2022: An ECMM Score to Measure QUALity of the Clinical Management of Invasive *Trichosporon* Infections. J. Antimicrob. Chemother..

[82] Gaurav V., Grover C., Das S., Rai G. (2022). White Piedra: An Uncommon Superficial Fungal Infection of Hair. Ski. Appendage Disord..

